# Supplementary material for: Bird clades with less complex appendicular skeletons tend to have higher species richness
Source: Nat Commun. 2023 Sep 19;14:5817. doi: 10.1038/s41467-023-41415-2 (PMC10509246; doi:10.1038/s41467-023-41415-2)
Supplement: Supplementary file 1 — Supplementary Information [file 41467_2023_41415_MOESM1_ESM.pdf]

Supplementary Information – Bird clades with less complex appendicular skeletons tend to be more diverse

Abbreviation	Institution
USNM + NMNH	National Museum of Natural history, Smithsonian Institution, Washington D.C., USA
NCSM	North Carolina Museum of Natural Sciences, Raleigh, North Carolina, USA
UMMZ	University of Michigan Museum of Zoology, Ann Arbor, Michigan, USA
MCZ	Museum of Comparative Zoology, Cambridge, Massachusetts, USA
YPM	Yale Peabody Museum, Yale University, Connecticut, USA
EP	Masaki Eda Collection, Hokkaido University Museum, Sapporo, Japan
HUMNH	Botanic Garden and Museum, Field Science Center for Northern Biosphere, Hokkaido University, Sapporo, Japan
KUGM	Department of Geology and Mineralogy, Kyoto University, Kyoto, Japan
MVZ	Museum of Vertebrate Zoology, University of California, Berkeley, California, USA
NMNSAS	Department of Zoology, National Museum of Nature and Science, Tsukuba, Japan
NMNSVP	Department of Geology and Paleontology, National Museum of Nature and Science, Tsukuba, Japan
YIO	Yamashina Institute for Ornithology, Abiko, Japan
LACM + NHMLAC	Ornithology Department, Natural History Museum of Los Angeles County, Los Angeles, California, USA
UWBM	University of Washington Burke Museum of Natural History and Culture, Seattle, Washington, USA
CAS	California Academy of Sciences, San Francisco, California, USA
AMB	Abiko City Museum of Birds, Abiko, Japan
NHMUK	Birds Collection, Natural History Museum, Tring, UK
MNHN	Muséum National d’Histoire Naturelle, Paris, France
MNHN-LAC	Muséum National d’Histoire Naturelle, Laboratoire d’Anatomie Comparée, Paris, France
MACN	Museo Argentino de Ciencias Naturales, Buenos Aires, Argentina

HUF	Laboratory of Marine Ecology, Division of Marine Bioresources and Environmental Science, Graduate School of Fisheries Sciences, Hokkaido University, Hakodate, Japan
CSIRO	CSIRO, Division of Wildlife Research, Canberra, Australia
AMNH	American Museum of Natural History, New York, USA
ZMUC	Zoological Museum, Copenhagen University, Copenhagen, Denmark
FMNH	Field Museum, Chicago, USA
MLP	Museo de La Plata, La Plata, Argentina
MNZ	Museum of New Zealand Te Papa Tongarewa, Wellington, New Zealand
UMZC	Museum of Zoology, University of Cambridge, Cambridge, UK

Supplementary Table 1. ***Institutional Abbreviations***. *Abbreviations for institutions whose collections provided specimens for the dataset provided in Supplementary Data 1, as stated in the "Spec_no." column.*

Supplementary Note 1 – Deriving an index of complexity applicable to the appendicular skeleton

Controlling for the impacts of body size variation upon our data was of critical importance. We could readily observe the correlation between bone lengths and estimates of body mass (Supplementary Figure 1).

However, standard protocols were not desirable in this case. It is typical for values with a large variance or skewed distribution, such as those observed for body mass and bone lengths in extant species, to be log-transformed prior to analysis. In this case, log-transformation would have produced an unwanted artefact of scaling, having a larger relative impact on species of larger size. Complexity is defined, in part, as differentiation within a structure. Species with the same relative proportions of limb bone lengths should, therefore, net equivalent complexity scores. We showed that isometrically-scaled models of the same relative limb bone proportions will yield different inferred complexity scores when they are $\log_{10}$-transformed prior to index calculation (Supplementary Table 8). Therefore, allowing size corrections to have different scaling effects across our sample of species was unacceptable, and the alternative method of isometric size transformation was used (see Methods in main text).

The chosen protocol involves expressing the length of each bone as a proportion of the mean length of all six for that species. This means that information on the relative length of each limb pair is retained during the transformation. To test this expectation, we implemented an alternative method, wherein the length of each bone was expressed as a proportion of the mean length for its respective limb pair. This was expected to remove contributions to complexity through relative differences in limb pair length entirely.

Comparison of complexity scores calculated from this method and the method detailed in Methods (main text) showed that both within- and between-limb pair differences in bone lengths are retained in the principal method (Supplementary Fig. 5). A 1:1 correlation line is indicated in that figure, along which some species can be observed to fall. This indicates that those species exhibit little or no difference in the total length of each limb pair, and that removing that variation has little impact on their inferred complexity scores. Conversely, many species diverge from that 1:1 correlation, indicating contributions from between-limb pair differences in total relative length. Therefore, it is clear that retaining those contributions is both necessary, and successfully achieved by the methods described in the Methods section in the main text.

Supplementary Figures

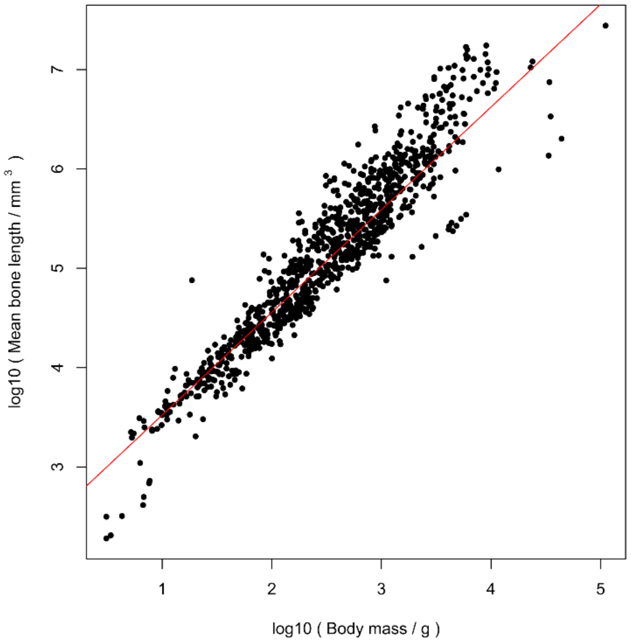

Supplementary Figure 1. ***A linear relationship between species body masses, and the cubed mean length of their limb bones.*** *This correlation clearly demonstrates the need to control for limb length variation related to variation in body size. Both body mass and cube of mean bone length were* log_{10}*-transformed to obtain data distributions appropriate for linear regression.*

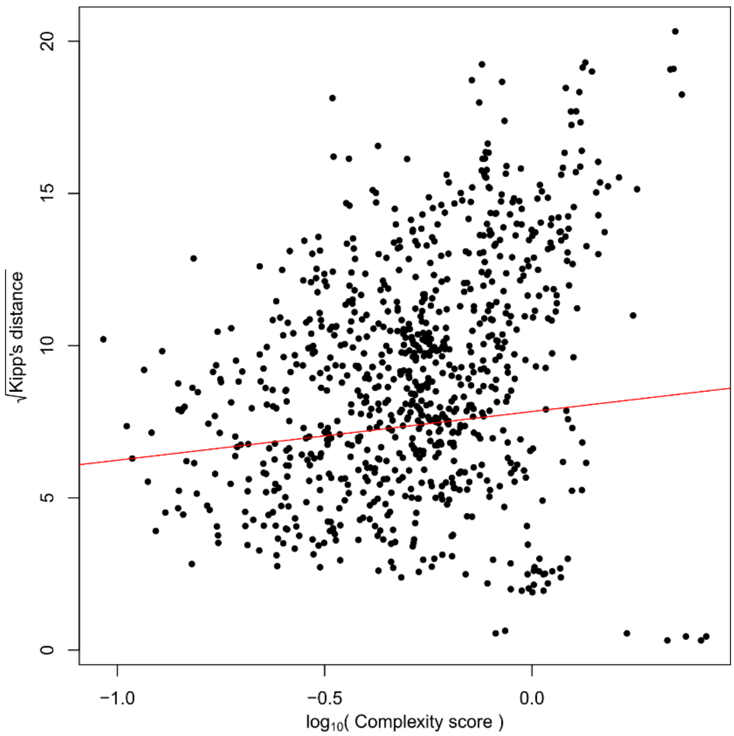

<u>Supplementary Figure 2.</u> ***Phylogenetic generalised least-squares (PGLS) analysis of complexity scores and Kipp's distances.*** *Although a positive correlation was recovered, the fit of the model is poor. It is observable that both the highest and lowest Kipp's distances occur for species in the sample with the greatest complexity scores, while intermediate Kipp's distances are associated with lower complexity.*

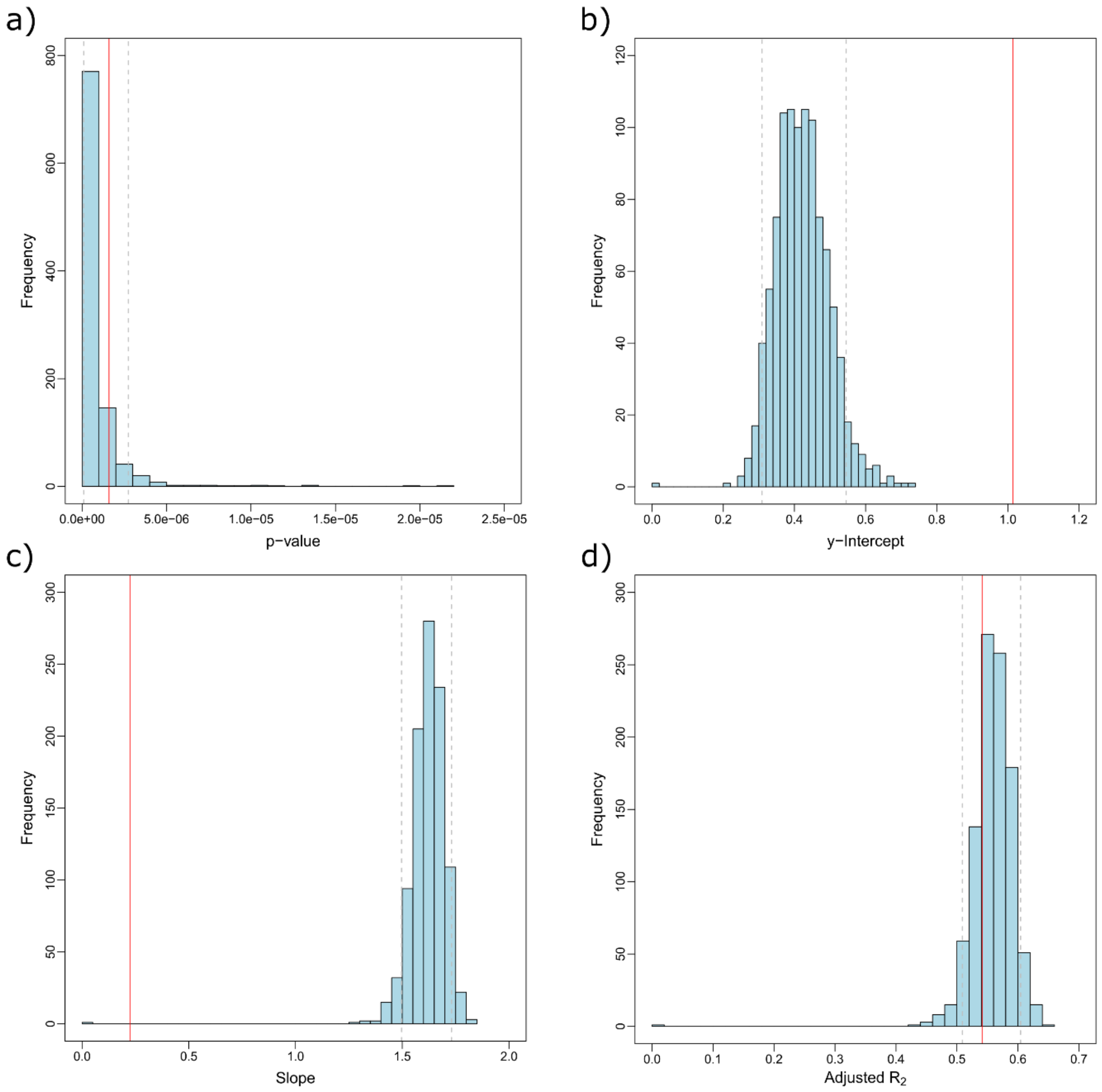

Supplementary Figure 3. ***Null distributions of model parameters from randomisation of foraging niche data.*** *Null distributions of a) p-values, b) y-intercept and c) slope coefficients, and d) adjusted* R^2*values obtained from refitting of phylogenetic generalised least-squares (PGLS) models to 1,000 randomised permutations of the foraging niche data. The 5% tails of the distributions are indicated with dashed grey lines, and values obtained from fitting the empirical PGLS model are indicated in red. The empirical p-value and adjusted* R^2 *were not significantly different from the null values, although the y-intercept was significantly greater and the slope significantly lower in the empirical model than the randomised ones. This was established using one-sided randomisation tests.*

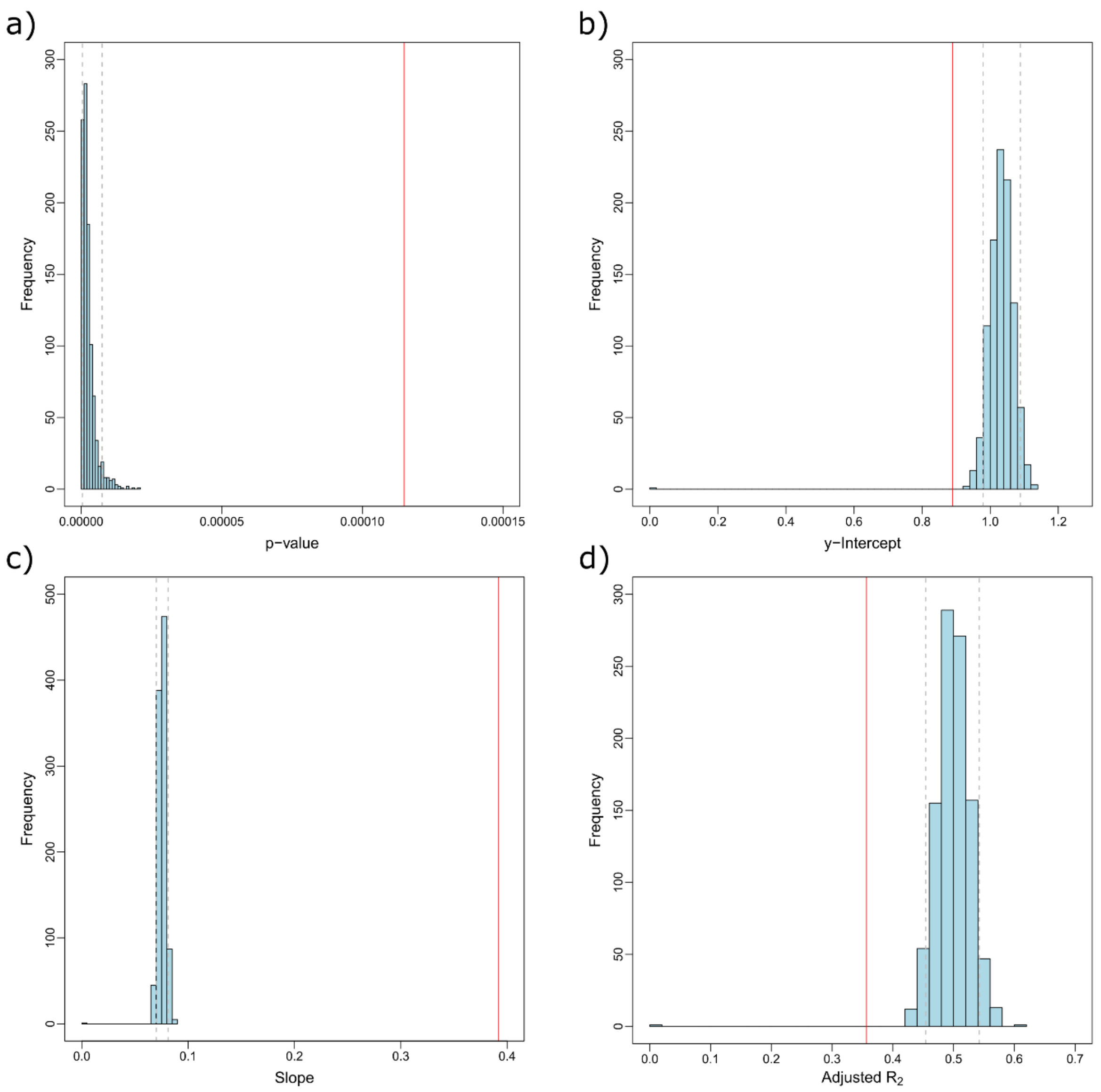

Supplementary Figure 4. ***Null distributions of model parameters from randomisation of trophic niche data.*** *Null distributions of a) p-values, b) y-intercept and c) slope coefficients, and d) adjusted R^2 values obtained from refitting of PGLS models to 1,000 randomised permutations of the trophic niche data. The 5% tails of the distributions are indicated with dashed grey lines, and values obtained from empirical model fitting are indicated in red. The empirical PGLS model returned a significantly greater p-value and significantly lower adjusted R^2 than the randomised models, as indicated by a one-sided randomisation test. The y-intercept of the empirical model was significantly lower than expected by chance, and the slope significantly greater.*

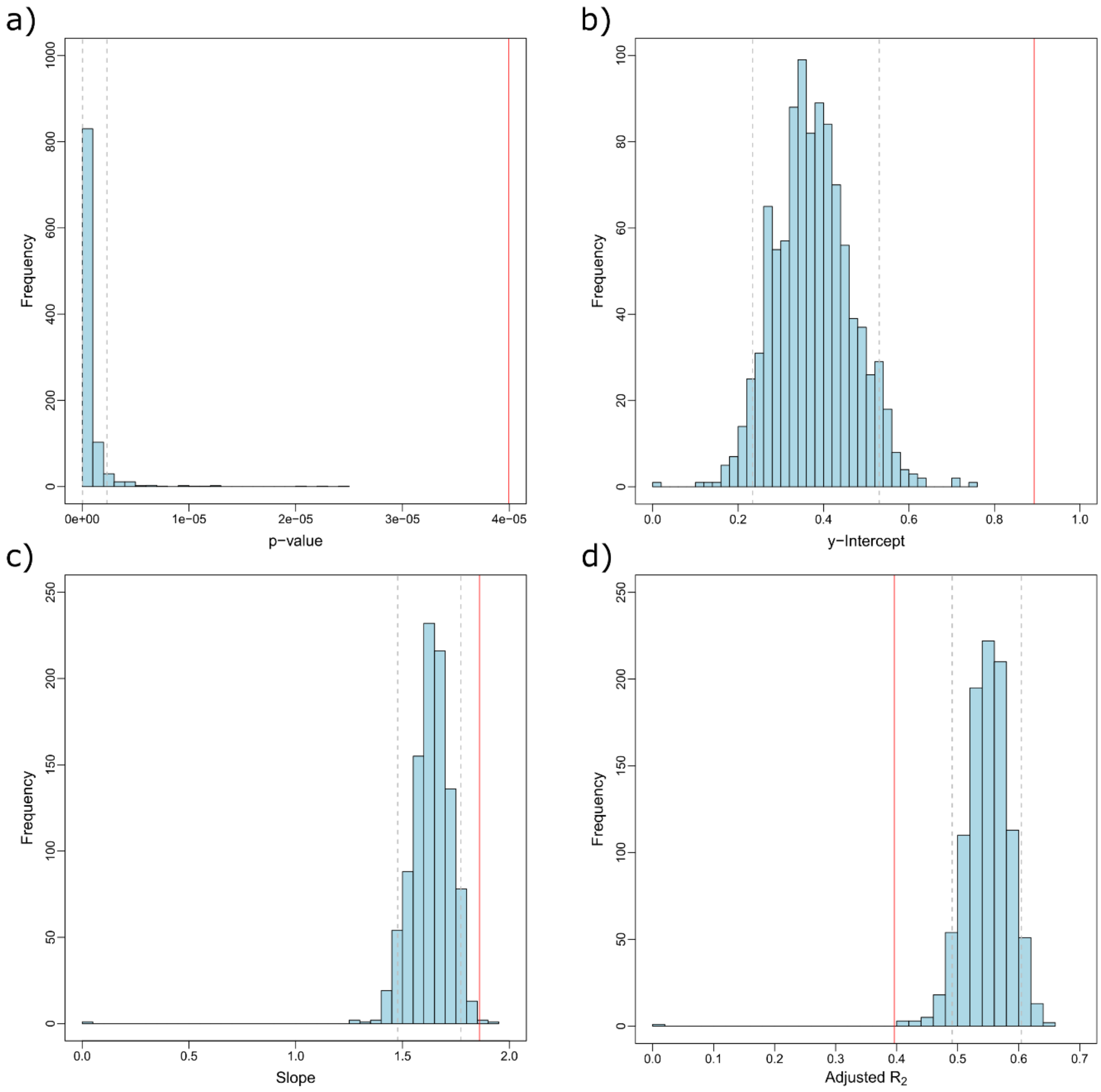

<u>Supplementary Figure 5.</u> ***Null distributions of model parameters from randomisation of habitat type data.*** *Null distributions of a) p-values, b) y-intercept and c) slope coefficients, and d) adjusted R^2values obtained from refitting of PGLS models to 1,000 randomised permutations of the habitat type data. The 5% tails of the distributions are indicated with dashed grey lines, and values obtained from empirical model fitting are indicated in red. The empirical PGLS model possessed a significantly greater p-value and significantly lower adjusted R^2 than the PGLS models fitted to randomised data, indicating a poorer fit for the empirical data. The y-intercept of the empirical model was significantly greater than expected by chance, as was the slope coefficient. These results were inferred using a one-sided randomisation test.*

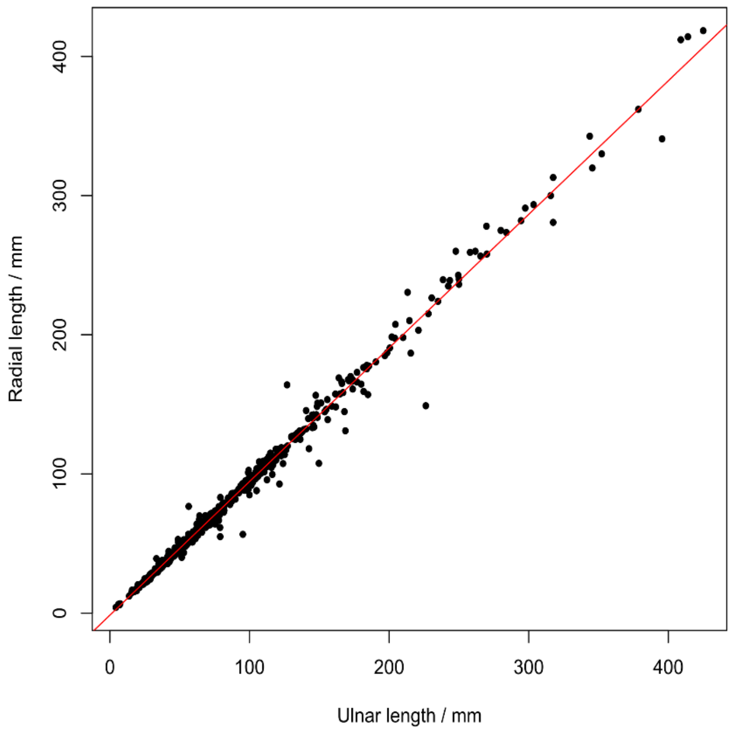

Supplementary Figure 6. ***Phylogenetic RMA of ulnar and radial lengths, measured in millimetres.*** *A strong positive correlation is clearly visible. This gives us confidence in our ability to infer missing ulnar lengths values from the length of the radius.*

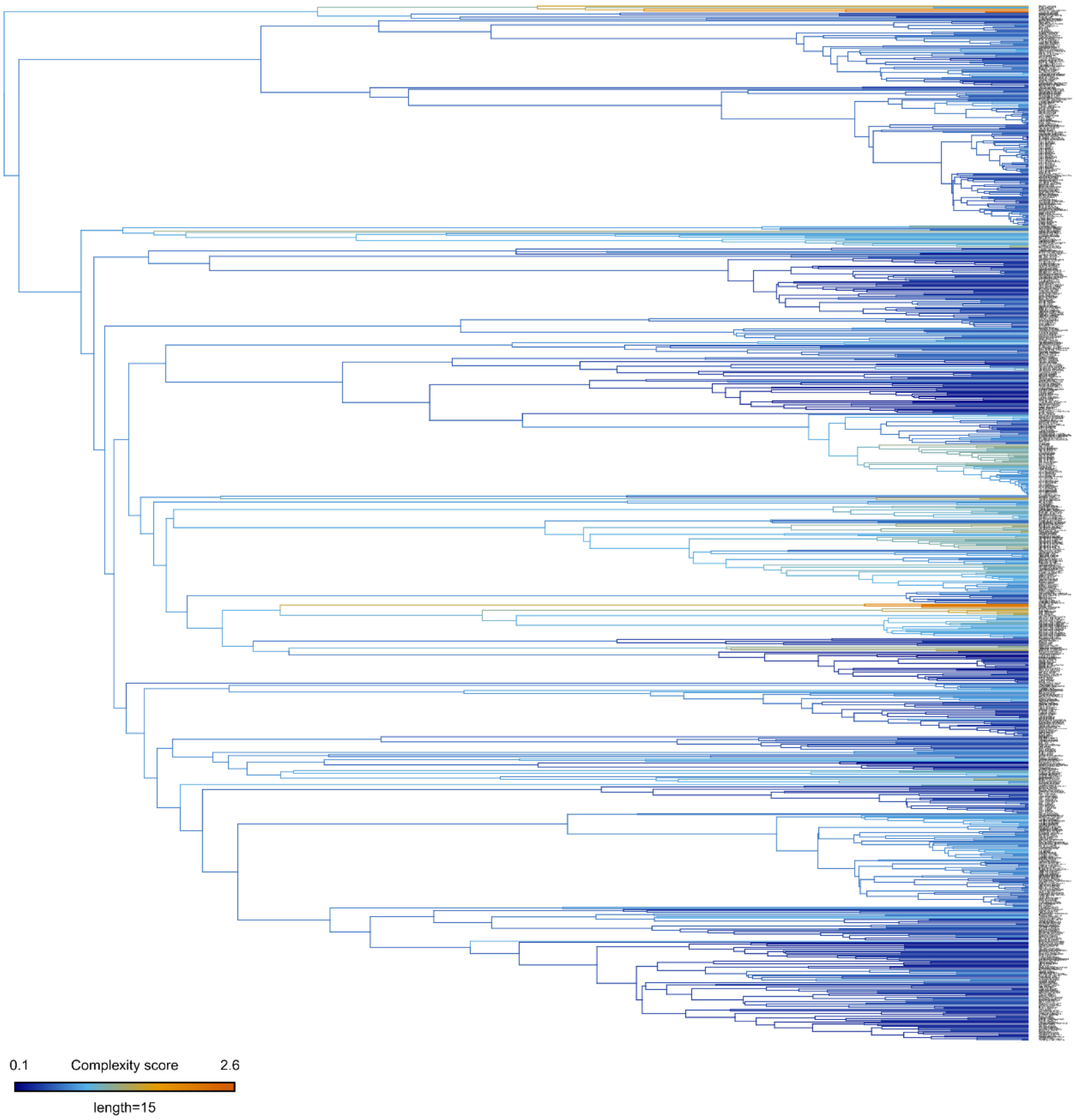

<u>Supplementary Figure 7.</u> ***The species-level phylogeny used in analyses.*** *Species complexity scores are indicated by the colour of the branches, with the lowest scores indicated in dark blue, and the greatest scores in orange. Values for internal branches are inferred from the tip data. The length of the legend bar corresponds to the branch lengths of the tree, and indicates a time span of 15 million years. A machine-readable copy of this tree is available in Supplementary Software.*

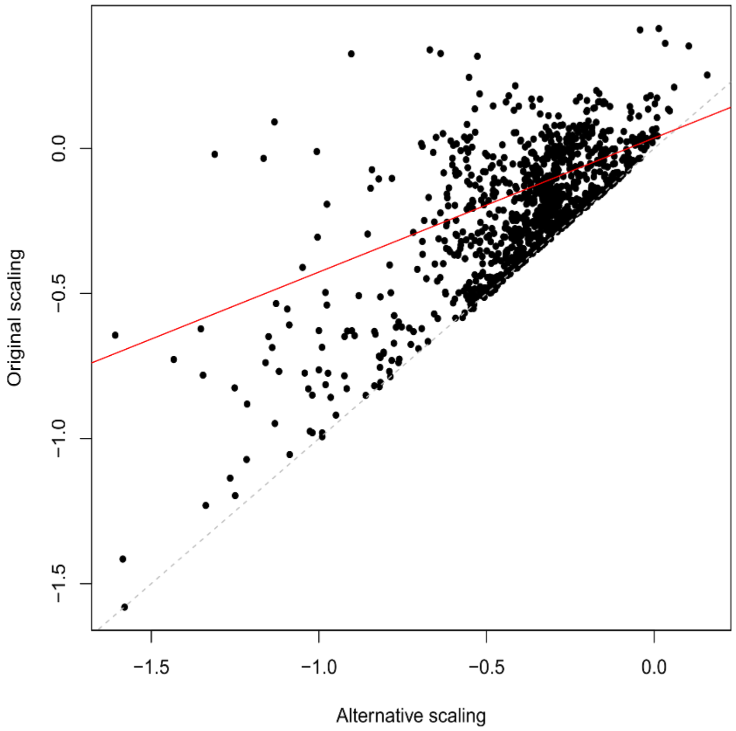

<u>Supplementary Figure 8.</u> ***A comparison of complexity scores derived using two methods of isometric transformation.*** *The original scaling, shown on the y-axis, was calculated using mean bone lengths expressed as a proportion of the mean of all six bones for that species. The alternative scaling, shown on the x-axis, expressed each length as a proportion of the mean length for that limb pair (either forelimb or hindlimb). Scores on both axes are* log_{10}*-transformed. The red line shows the recovered correlation from a PGLS analysis of these scores, and the dashed grey line indicates a perfect 1:1 correlation. This demonstrates that variation in limb pair lengths contributes to a greater inferred complexity score in some species, but not in others. Correction for allometric variation was not performed for the purpose of this demonstration.*

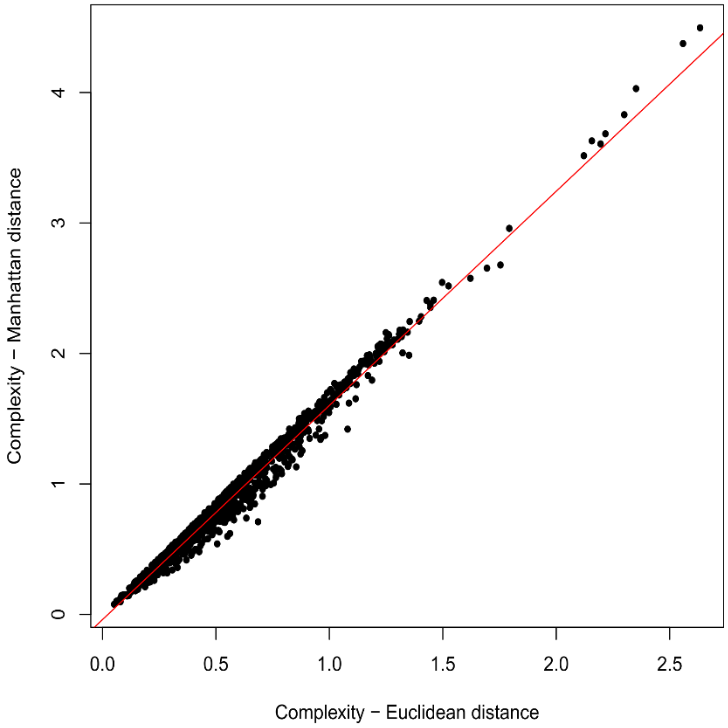

Supplementary Figure 9. ***Correlation obtained from PGLS regression of complexity scores derived using Euclidean versus Manhattan distance.*** *The strength of the linear correlation gives confidence that the choice of distance metric does not have substantial impact upon results and their interpretation. The recovered trend line is indicated in red.*

Supplementary Tables

**Mean complexity score per foraging niche**	Complexity – Euclidean distance	Complexity – Manhattan distance	Alt Complexity – Euclidean distance	Alt Complexity – Manhattan distance
Aquatic dive	0.787625	1.062719	0.660445	1.030552
Herbivore aquatic surface	0.702417	0.921966	0.60174	0.997885
Aquatic surface	1.027919	1.424517	0.925868	1.512969
Aquatic aerial	1.159194	1.772053	1.151072	1.892607
Herbivore ground	0.651403	0.858016	0.510153	0.85246
Invertivore ground	0.869042	1.335619	0.542026	0.852369
Vertivore air to surface	0.445273	0.668819	0.454604	0.675403
Aquatic ground	0.695152	1.105736	0.370066	0.568252
Frugivore glean	0.3410	0.523654	0.501793	0.75327
Invertivore glean arboreal	0.767146	1.132678	0.38062	0.593959
Herbivore aquatic dive	0.686414	0.889991	0.574947	0.954758
Scavenger ground	0.826114	1.217188	0.758833	1.207132
Invertivore bark	0.543147	0.803848	0.414955	0.624562
Aquatic perch	0.782229	1.253885	0.917813	1.434999
Frugivore aerial	0.677075	1.047277	0.710848	1.035924
Invertivore aerial	0.61478	0.974703	0.809576	1.250701
Generalist	0.701119	0.975314	0.613937	0.990975
Invertivore sally air	0.46777	0.727563	0.543701	0.853326
Invertivore sally ground	0.522915	0.778666	0.573741	0.91355
Invertivore sally surface	0.515464	0.755151	0.478778	0.723306
Aquatic plunge	1.123271	1.712598	1.163744	1.932413
Granivore ground	0.33783	0.502853	0.419755	0.629667
Herbivore aquatic ground	0.927163	1.352227	0.615825	0.954999
Vertivore perch	0.447776	0.668447	0.354011	0.526188
Frugivore ground	1.055057	1.640858	0.968695	1.647778
Vertivore ground	0.791061	1.290589	0.578716	0.847918
Vertivore aerial	0.205902	0.309309	0.364547	0.573083
Granivore arboreal	0.516844	0.810313	0.718642	1.059653
Nectarivore glean	0.483322	0.705294	0.602579	0.902415
Nectarivore aerial	1.167304	1.785156	1.006662	1.668337

Supplementary Table 2. ***The mean complexity index for species occupying each of the 30 sampled foraging niche categories.*** *"Alt Complexity" refers to the complexity scores used in all analyses, for which allometric variation has been properly controlled for. The non-“Alt” scores refer to scores calculated directly from isometrically-transformed mean bone lengths, without additional modelling to remove allometric variation in lengths. These were not used in analyses. Complexity scores derived using both Euclidean and Manhattan distances are provided.*

**Mean complexity score per trophic niche**	Complexity – Euclidean distance	Complexity – Manhattan distance	Alt Complexity – Euclidean distance	Alt Complexity – Manhattan distance
Aquatic predator	0.868714	1.267265	0.71937	1.151143
Omnivore	0.646933	0.969549	0.542749	0.842483
Herbivore aquatic	0.724397	0.962087	0.596753	0.98466
Herbivore terrestrial	0.636494	0.846075	0.498008	0.823813
Invertivore	0.715562	1.090702	0.544748	0.849019
Vertivore	0.435689	0.654688	0.393737	0.587967
Frugivore	0.461969	0.713536	0.570285	0.881546
Scavenger	0.826114	1.217188	0.758833	1.207132
Granivore	0.384729	0.582899	0.46896	0.701593
Nectarivore	0.794835	1.196993	0.784698	1.251827

Supplementary Table 3. ***The mean complexity index for species occupying each of the 10 sampled trophic niche categories.*** *"Alt Complexity" refers to the complexity scores used in all analyses, for which allometric variation has been properly controlled for. The non-"Alt" scores refer to scores calculated directly from isometrically-transformed mean bone lengths.*

**Mean complexity score per habitat type**	Complexity – Euclidean distance	Complexity – Manhattan distance	Alt Complexity – Euclidean distance	Alt Complexity – Manhattan distance
Wetland	0.734983	1.05234	0.514985	0.816345
Marine	1.006804	1.438309	0.947696	1.54281
Forest	0.575314	0.875459	0.561446	0.868226
Grassland	0.595928	0.89295	0.468039	0.724216
Shrubland	0.69268	1.046465	0.540725	0.839809
Woodland	0.512923	0.781922	0.507454	0.760489
Human modified	0.556426	0.822701	0.481499	0.753818
Riverine	0.738222	1.090319	0.63616	1.013404
Coastal	0.736479	1.09557	0.601519	0.979826
Rock	0.690304	1.045011	0.575464	0.87732
Desert	0.640705	0.891645	0.301505	0.432192

Supplementary Table 4. ***The mean complexity index for species occupying each of the 11 sampled habitat type categories.*** *"Alt Complexity" refers to the complexity scores used in all analyses, for which allometric variation has been properly controlled for. The non-"Alt" scores refer to scores calculated directly from isometrically-transformed mean bone lengths, which would still be expected to contain some signal of body size variation. Note, the "Desert" category is only represented in this case by a single member.*

**PGLS - Species richness ~ Mean complexity**	Complexity measured using Euclidean distance	Complexity measured using Manhattan distance
$\log_{10}$ (Mean complexity score)<br>- Isometric transformation	p = 0.0134<br>F = 6.86 on 1 & 32 d.f.<br>Adj.R^2 = 0.1508<br>Intercept = 1.450<br>Slope = -1.565<br>k = 2.259	p = 0.0187<br>F = 6.139 on 1 & 32 d.f.<br>Adj.R^2 = 0.135<br>Intercept = 1.749<br>Slope = -1.422<br>k = 2.163
$\log_{10}$ (Mean complexity score)<br>- $\log_{10}$-transformation	p = 0.0165<br>F = 6.407 on 1 & 32 d.f.<br>Adj.R^2 = 0.1408<br>Intercept = 0.943<br>Slope = -1.527<br>k = 2.373	p = 0.0222<br>F = 5.777 on 1 & 32 d.f.<br>Adj.R^2 = 0.1265<br>Intercept = 1.293<br>Slope = -1.387<br>k = 2.274
$\log_{10}$ (Mean complexity score)<br>- Isometric transformation<br>- No Passeriformes	p = 0.0242<br>F = 5.621 on 1 & 31 d.f.<br>Adj.R^2 = 0.1262<br>Intercept = 1.443<br>Slope = -1.313<br>k = 2.317	p = 0.0346<br>F = 4.886 on 1 & 31 d.f.<br>Adj.R^2 = 0.1083<br>Intercept = 1.703<br>Slope = -1.178<br>k = 2.208

Supplementary Table 5. ***PGLS model results from pairwise comparisons of mean clade complexity and clade species richness.*** *Both variables were $\log_{10}$-transformed prior to analysis. Phylogenetic generalised least-squares (PGLS) results are displayed for models fitted using complexity scores derived using both Euclidean and Manhattan distances (columns), as described in Methods. Further PGLS results are shown for an alternative method using an initial $\log_{10}$-transformation of mean bone lengths in place of the isometric scaling method described in Methods (rows). All have been subsequently controlled for allometry prior to calculation of complexity scores. The identifiers "Isometric" and "$\log_{10}$" refer only to the first step of data processing. The Euclidean distance metric was used for this sensitivity analysis. Also shown are results obtained from PGLS models fitted to the Euclidean and Manhattan distance data described in Methods, with the Order Passeriformes excluded as an outlier. Results are congruent across approaches.*

**PGLS - Complexity ~ ecological niches or body mass**	log_{10} (body mass / g)	Ecology – foraging niche	Ecology – trophic niche	Ecology – habitat type	Kipp's distance
log_{10} (Complexity scores)<br>- Euclidean distance<br>- Isometric	p = 0.3024<br>F = 1.065 on 1 & 981 d.f.<br>Adj.R^2 = 6.588_{x10}^{-5}<br>Intercept = -0.249<br>Slope = 0.0148<br>k = 0.661	p = 3.359_{x10}^{-4}<br>F = 2.192 on 29 & 743 d.f.<br>Adj.R^2 = 0.0429<br>k = 0.698	p = 6.504_{x10}^{-5}<br>F = 3.928 on 9 & 860 d.f.<br>Adj.R^2 = 0.0294<br>k = 0.703	p = 0.0213<br>F = 2.182 on 9 & 858 d.f.<br>Adj.R^2 = 0.0121<br>k = 0.689	p = 4.275_{x10}^{-5}<br>F = 16.920 on 1 & 859 d.f.<br>Adj.R^2 = 0.0182<br>Intercept = 7.826<br>Slope = 1.599<br>k = 0.587
log_{10} (Complexity score)<br>- Manhattan distance<br>- Isometric	p = 0.326<br>F = 0.965 on 1 & 981 d.f.<br>Adj.R^2 = -3.562_{x10}^{-5}<br>Intercept = -0.0524<br>Slope = 0.0150<br>k = 0.644	p = 0.0462<br>F = 1.499 on 29 & 743 d.f.<br>Adj.R^2 = 0.0208<br>k = 0.729	p = 3.097_{x10}^{-3}<br>F = 2.939 on 9 & 860 d.f.<br>Adj.R^2 = 0.0223<br>k = 0.717	p = 0.0191<br>F = 2.222 on 9 & 858 d.f.<br>Adj.R^2 = 0.0159<br>k = 0.705	p = 6.402_{x10}^{-5}<br>F = 16.140 on 1 & 859 d.f.<br>Adj.R^2 = 0.0173<br>Intercept = 7.522<br>Slope = 1.465<br>k = 0.589
log_{10} (Complexity scores)<br>- Euclidean distance<br>- log_{10}	p = 0.7144<br>F = 0.134 on 1 & 981 d.f.<br>Adj.R^2 = 8.826_{x10}^{-4}<br>Intercept = -0.5989<br>Slope = -0.0086<br>k = 0.116	p = 0.0553<br>F = 1.478 on 29 & 743 d.f.<br>Adj.R^2 = 0.0176<br>k = 0.688	p = 6.504_{x10}^{-5}<br>F = 3.928 on 9 & 860 d.f.<br>Adj.R^2 = 0.0294<br>k = 0.703	p = 0.0395<br>F = 1.973 on 9 & 858 d.f.<br>Adj.R^2 = 0.010<br>k = 0.741	p = 2.562_{x10}^{-7}<br>F = 26.990 on 9 & 858 d.f.<br>Adj.R^2 = 0.0293<br>k = 0.600

Supplementary Table 6. ***PGLS model results obtained from pairwise comparison of species complexity scores against body mass and foraging, trophic, and habitat niches, respectively.*** *Also shown are results from PGLS modelling of complexity scores against estimates of body mass. Results are displayed for models fitted using complexity scores derived using both Euclidean and Manhattan distances, as described in Methods. Also shown are PGLS results obtained from an alternative method of calculating complexity scores, which used an initial log_{10}-transformation of mean bone lengths in place of the isometric scaling method described in Methods. These are indicated with "log_{10}" and "Isometric" identifiers, respectively. Results are congruent across approaches.*

**PGLS - Mean ordinal complexity or species richness ~ No. of occupied ecological niches**	Number of occupied foraging niches	Number of occupied trophic niches	$\log_{10}$ (Number of occupied habitat types)
$\log_{10}$(Mean complexity score)<br>- Euclidean distance metric<br>- Isometric	$p = 5.779_{x10}^{-3}$<br>F = 8.834 on 1 & 30 d.f.<br>Adj.R^2 = 0.2017<br>Intercept = -0.1110<br>Slope = -0.0314<br>k = 2.250	p = 0.3518<br>F = 0.893 on 1 & 32 d.f.<br>Adj.R^2 = -3.254_{x10}^{-3}<br>Intercept = -0.1551<br>Slope = -0.0318<br>k = 1.534	p = 0.0645<br>F = 3.668 on 1 & 32 d.f.<br>Adj.R^2 = 0.0748<br>Intercept = -0.0859<br>Slope = -0.2848<br>k = 1.470
$\log_{10}$(Mean complexity score)<br>- Manhattan distance metric<br>- Isometric	$p = 7.524_{x10}^{-3}$<br>F = 8.215 on 1 & 30 d.f.<br>Adj.R^2 = 0.1888<br>Intercept = 0.0833<br>Slope = -0.0320<br>k = 2.023	p = 0.3533<br>F = 0.8872 on 1 & 32 d.f.<br>Adj.R^2 = -3.431_{x10}^{-3}<br>Intercept = 0.0386<br>Slope = -0.0329<br>k = 1.535	p = 0.0908<br>F = 3.041 on 1 & 32 d.f.<br>Adj.R^2 = 0.0582<br>Intercept = 0.0992<br>Slope = -0.2721<br>k = 1.471
$\log_{10}$(Mean complexity score)<br>- Euclidean distance metric<br>- $\log_{10}$	$p = 5.014_{x10}^{-3}$<br>F = 9.173 on 1 & 30 d.f.<br>Adj.R^2 = 0.2086<br>Intercept = -0.4547<br>Slope = -0.0296<br>k = 2.320	p = 0.4279<br>F = 0.6448 on 1 & 32 d.f.<br>Adj.R^2 = -0.0109<br>Intercept = -0.5030<br>Slope = -0.0259<br>k = 1.666	p = 0.0855<br>F = 3.148 on 1 & 32 d.f.<br>Adj.R^2 = 0.0611<br>Intercept = -0.4351<br>Slope = -0.2543<br>k = 1.560
$\log_{10}$ (Species richness)	$p = 9.680_{x10}^{-7}$<br>F = 37.590 on 1 & 30 d.f.<br>Adj.R^2 = 0.5413<br>Intercept = 1.0146<br>Slope = 0.2238<br>k = 1.809	$p = 1.149_{x10}^{-4}$<br>F = 19.30 on 1 & 32 d.f.<br>Adj.R^2 = 0.3567<br>Intercept = 0.8897<br>Slope = 0.3919<br>k = 1.275	$p = 3.994_{x10}^{-5}$<br>F = 22.650 on 1 & 32 d.f.<br>Adj.R^2 = 0.3962<br>Intercept = 0.8928<br>Slope = 1.8602<br>k = 2.088

Supplementary Table 7. ***PGLS model results from comparison of clade mean complexity and species richness, and the number of occupied ecological niches.*** *Results are provided for PGLS analyses including foraging, trophic, and habitat niches. Results are displayed for PGLS models fitted using complexity scores derived using both Euclidean and Manhattan distances, as described in Methods, as well as results obtained from an alternative method using an initial $\log_{10}$-transformation of mean bone lengths. This is distinguished with the identifier "$\log_{10}$", as opposed to "Isometric". Results are consistent across approaches, with the exception of the marginal significance of the correlation between mean complexity and the number of occupied habitat niches being lost.*

**Additive PGLS – Species Richness ~ Mean complexity + No. of occupied niches**	Number of occupied foraging niches	Number of occupied trophic niches	$\log_{10}$ (Number of occupied habitat types)
$\log_{10}$(Mean complexity score)<br>- Euclidean distance<br>- Isometric transformation	p = 1.499_{x10}^{-6}<br>F = 22.060 on 2 & 29 d.f.<br>Adj.R^2 = 0.5761<br>Intercept = 0.9030<br>Slope (category number) = 0.1974<br>p (category number) = 4.604_{x10}^{-6}<br>Slope (mean complexity) = -0.8899<br>p (mean complexity) = 0.0734	p = 3.365_{x10}^{-5}<br>F = 14.620 on 2 & 31 d.f.<br>Adj.R^2 = 0.4523<br>Intercept = 0.7330<br>Slope (category number) = 0.3363<br>p (category number) = 2.213_{x10}^{-4}<br>Slope (mean complexity) = -1.2482<br>p (mean complexity) = 5.616_{x10}^{-3}	p = 2.192_{x10}^{-5}<br>F = 15.470 on 2 & 31 d.f.<br>Adj.R^2 = 0.4672<br>Intercept = 0.7679<br>Slope (category number) = 1.6049<br>p (category number) = 1.568_{x10}^{-4}<br>Slope (mean complexity) = -0.9884<br>p (mean complexity) = 0.0249
$\log_{10}$(Mean complexity score)<br>- Manhattan distance<br>- Isometric transformation	p = 1.673_{x10}^{-6}<br>F = 21.790 on 2 & 29 d.f.<br>Adj.R^2 = 0.5728<br>Intercept = 1.0682<br>Slope (category number) = 0.1995<br>p (category number) = 3.707_{x10}^{-5}<br>Slope (mean complexity) = -0.8084<br>p (mean complexity) = 0.0835	p = 4.445_{x10}^{-5}<br>F = 14.090 on 2 & 31 d.f.<br>Adj.R^2 = 0.4423<br>Intercept = 0.9698<br>Slope (category number) = 0.3397<br>p (category number) = 2.203_{x10}^{-4}<br>Slope (mean complexity) = -1.1417<br>p (mean complexity) = 8.9024_{x10}^{-3}	p = 1.934_{x10}^{-5}<br>F = 15.720 on 2 & 31 d.f.<br>Adj.R^2 = 0.4715<br>Intercept = 0.9407<br>Slope (category number) = 1.6380<br>p (category number) = 1.029_{x10}^{-4}<br>Slope (mean complexity) = -0.9645<br>p (mean complexity) = 0.0216
$\log_{10}$(Mean complexity score)<br>- Euclidean distance<br>- $\log_{10}$ transformation	p = 1.916_{x10}^{-6}<br>F = 21.450 on 2 & 29 d.f.<br>Adj.R^2 = 0.5688<br>Intercept = 0.6114<br>Slope (category number) = 0.2003<br>p (category number) = 4.533_{x10}^{-5}<br>Slope (mean complexity) = -0.8556<br>p (mean complexity) = 0.0977	p = 4.681_{x10}^{-5}<br>F = 13.990 on 2 & 31 d.f.<br>Adj.R^2 = 0.4405<br>Intercept = 0.3228<br>Slope (category number) = 0.3421<br>p (category number) = 2.084_{x10}^{-4}<br>Slope (mean complexity) = -1.2294<br>p (mean complexity) = 9.8026_{x10}^{-3}	p = 2.565_{x10}^{-5}<br>F = 15.160 on 2 & 31 d.f.<br>Adj.R^2 = 0.4618<br>Intercept = 0.4231<br>Slope (category number) = 1.6252<br>p (category number) = 1.364_{x10}^{-4}<br>Slope (mean complexity) = -1.0104<br>p (mean complexity) = 0.0298

Supplementary Table 8. ***PGLS results obtained from additive models of mean clade complexity and the number of occupied niches in combination.*** *In each case, the number of occupied foraging, trophic, or habitat are used to explain clade species richness alongside mean complexity, respectively. PGLS models were fitted using complexity scores derived using both Euclidean and Manhattan distances, as described in Methods. Further PGLS results are shown for an alternative method using an initial $\log_{10}$-transformation of mean bone lengths. These distinctions are noted with the identifiers "$\log_{10}$" and "Isometric" to indicate the first step, and "Euclidean" or "Manhattan" to denote the distance metric used.*

**Scale factor**	**0.1**	**0.25**	**0.5**	**1**	**1.5**	**2**
Humerus	5	12.5	25	50	75	100
Ulna	6	15	30	60	90	120
Carpometacarpus	3.5	8.75	17.5	35	52.5	70
Femur	6	15	30	60	90	120
Tibiotarsus	9	22.5	45	90	135	180
Tarsometatarsus	7	17.5	35	70	105	140
**Scale factor**	**0.1**	**0.25**	**0.5**	**1**	**1.5**	**2**
$\log_{10}$ (Humerus)	0.9120	0.9420	0.9540	0.9618	0.9653	0.9674
$\log_{10}$ (Ulna)	1.0153	1.0101	1.0080	1.0066	1.0060	1.0057
$\log_{10}$ (Carpometacarpus)	0.7099	0.8090	0.8482	0.8741	0.8855	0.8924
$\log_{10}$ (Femur)	1.0153	1.0101	1.0080	1.0066	1.0060	1.0057
$\log_{10}$ (Tibiotarsus)	1.2450	1.1613	1.1282	1.1063	1.0967	1.0908
$\log_{10}$ (Tarsometatarsus)	1.1026	1.0675	1.0537	1.0445	1.0405	1.0380
**Complexity score**	**0.4666**	**0.3071**	**0.2440**	**0.2025**	**0.1841**	**0.1730**

Supplementary Table 9. ***Proof that a log-transformation of raw values results in differing complexity scores.*** *This shows the undesirable effects of log-transformation using isometrically-scaled models of the same proportional differences in limb bone lengths**.** Simulated lengths for the studied skeletal elements and their respective scale factor are indicated in the top section of the table. The bottom section of the table shows these same scaled lengths following a* $\log_{10}$ *transformation, and details the differing complexity scores implied by each set of measurements.*

Supplementary References

Full references for the publications listed in the "Reference" column of Supplementary Data 1.

1. Bell, A. *et al.* Quantitative analysis of morphometric data of pre-modern birds: Phylogenetic versus ecological signal. *Frontiers in Earth Science* **9**, doi:10.3389/feart.2021.663342 (2021).
2. Berger, A. J. The comparative functional morphology of the pelvic appendage in three genera of Cuculidae. *The American Midland Naturalist* **47**, 513-605, doi:10.2307/2422033 (1952).
3. Bochenski, Z. & Bochenski, Z. M. An Old World hummingbird from the Oligocene: a new fossil from Polish Carpathians. *J. Ornithol.* **149**, 211-216, doi:10.1007/s10336-007-0261-y (2008).
4. Böhmer, C., Plateau, O., Cornette, R. & Abourachid, A. Correlated evolution of neck length and leg length in birds. *R. Soc. Open Sci.* **6**, 13, doi:10.1098/rsos.181588 (2019).
5. Bourdon, E., Kristoffersen, A. V. & Bonde, N. A roller-like bird (Coracii) from the Early Eocene of Denmark. *Scientific Reports* **6**, 34050, doi:10.1038/srep34050 (2016).
6. Degrange, F. J., Noriega, J. I. & Vizcaíno, S. F. Morphology of the forelimb of *Psilopterus bachmanni* (Aves, Cariamiformes) (Early Miocene of Patagonia). *Paläontologische Zeitschrift* **89**, 1087-1096, doi:10.1007/s12542-015-0269-1 (2015).
7. Degrange, F. J. & Tambussi, C. P. Re-examination of *Psilopterus lemoinei* (Aves, Phorusrhacidae), a Late Early Miocene little terror bird from Patagonia (Argentina). *Journal of Vertebrate Paleontology* **31**, 1080-1092 (2011).
8. Field, D. J. & Hsiang, A. Y. A North American stem turaco, and the complex biogeographic history of modern birds. *BMC Evolutionary Biology* **18**, 102, doi:10.1186/s12862-018-1212-3 (2018).
9. Field, D. J., Lynner, C., Brown, C. & Darroch, S. A. F. Skeletal correlates for body mass estimation in modern and fossil flying birds. *PLoS One* **8**, e82000, doi:10.1371/journal.pone.0082000 (2013).
10. Fisher, H. I. Adaptations and comparative anatomy of the locomotor apparatus of New World vultures. *The American Midland Naturalist* **35**, 545-727 (1946).
11. Gatesy, S. M. & Middleton, K. M. Bipedalism, flight, and the evolution of theropod locomotor diversity. *Journal of Vertebrate Paleontology* **17**, 308-329, doi:10.1080/02724634.1997.10010977 (1997).

12. Hinić-Frlog, S. & Motani, R. Relationship between osteology and aquatic locomotion in birds: determining modes of locomotion in extinct Ornithurae. *J. Evol. Biol.* **23**, 372-385, doi:10.1111/j.1420-9101.2009.01909.x (2010).
13. James, H. F. & Olson, S. L. Descriptions of thirty-two new species of birds from the Hawaiian Islands: Part II. Passeriformes. *Ornithological Monographs*, 1-88, doi:10.2307/40166713 (1991).
14. Li, Z., Zhou, Z., Deng, T., Li, Q. & Clarke, J. A. A falconid from the Late Miocene of northwestern China yields further evidence of transition in Late Neogene steppe communities. *The Auk* **131**, 335-350 (2014).
15. Livezey, B. C. Morphology of flightlessness in *Chendytes*, fossil seaducks (Anatidae: Mergini) of Coastal California. *Journal of Vertebrate Paleontology* **13**, 185-199 (1993).
16. Manegold, A., Pavia, M. & Haarhoff, P. A new species of *Aegypius* vulture (Aegypiinae, Accipitridae) from the early Pliocene of South Africa. *Journal of Vertebrate Paleontology* **34**, 1394-1407, doi:10.1080/02724634.2014.863204 (2014).
17. Mayr, G. The middle Eocene European “ratite” *Palaeotis* (Aves, Palaeognathae) restudied once more. *Paläontologische Zeitschrift* **89**, 503-514, doi:10.1007/s12542-014-0248-y (2015).
18. Middleton, K. M. & Gatesy, S. M. Theropod forelimb design and evolution. *Zoological Journal of the Linnean Society* **128**, 149-187, doi:https://doi.org/10.1006/zjls.1998.0193 (2000).
19. Millener, P. R. & Worthy, T. H. Contributions to New Zealand's Late Quaternary avifauna. II: *Dendroscansor decurvirostris*, a new genus and species of wren (Aves: Acanthisittidae). *Journal of the Royal Society of New Zealand* **21**, 179-200, doi:10.1080/03036758.1991.10431406 (1991).
20. Norberg, U. M. Morphology of the wings, legs and tail of three coniferous forest tits, the goldcrest, and the treecreeper in relation to locomotor pattern and feeding station selection. *Philosophical Transactions of the Royal Society of London. B, Biological Sciences* **287**, 131-165, doi:doi:10.1098/rstb.1979.0054 (1979).
21. Olson, S. L. An early Eocene oilbird from the Green River Formation of Wyoming (Caprimulgiformes: Steatornithidae). *Documents des Laboratoires de Géologie de Lyon* **99**, 57-69 (1987).
22. Owre, O. T. Adaptations for locomotion and feeding in the anhinga and double-crested cormorant. *Ornithological Monographs* **6**, 1-138 (1967).

23. Smith, N. A. & Clarke, J. A. An alphataxonomic revision of extinct and extant razorbills (Aves, Alcidae): A combined morphometric and phylogenetic approach. *Ornithological Monographs* **72**, 1-61, doi:10.1525/om.2011.72.1.1 (2011).
24. Watanabe, J. Clade-specific evolutionary diversification along ontogenetic major axes in avian limb skeleton. *Evolution* **72**, 2632-2652, doi:10.1111/evo.13627 (2018).
25. Worthy, T. H. Two late-Glacial avifaunas from eastern North Island, New Zealand - Te Aute Swamp and Wheturau Quarry. *Journal of the Royal Society of New Zealand* **30**, 1-25, doi:10.1080/03014223.2000.9517607 (2000).
26. Zeffer, A., Johansson, L. C. & Marmebro, Å. Functional correlation between habitat use and leg morphology in birds (Aves). *Biol. J. Linnean Soc.* **79**, 461-484, doi:10.1046/j.1095-8312.2003.00200.x (2003).
27. Zhang, Z., Zheng, X., Zheng, G. & Hou, L. A new Old World vulture (Falconiformes: Accipitridae) from the Miocene of Gansu Province, northwest China. *J. Ornithol.* **151**, 401-408, doi:10.1007/s10336-009-0468-1 (2010).
