# Supplementary material for: Bird clades with less complex appendicular skeletons tend to have higher species richness
Source: Nat Commun. 2023 Sep 19;14:5817. doi: 10.1038/s41467-023-41415-2 (PMC10509246; doi:10.1038/s41467-023-41415-2)
Supplement: Supplementary file 3 — Reporting Summary [file 41467_2023_41415_MOESM3_ESM.pdf]

nature portfolio

Corresponding author(s): Andrew Brinkworth

Last updated by author(s): 14/8/23

Reporting Summary

Nature Portfolio wishes to improve the reproducibility of the work that we publish. This form provides structure for consistency and transparency in reporting. For further information on Nature Portfolio policies, see our Editorial Policies and the Editorial Policy Checklist.

Statistics

For all statistical analyses, confirm that the following items are present in the figure legend, table legend, main text, or Methods section.

n/a	Confirmed	
☐	☒	The exact sample size (*n*) for each experimental group/condition, given as a discrete number and unit of measurement
☐	☒	A statement on whether measurements were taken from distinct samples or whether the same sample was measured repeatedly
☐	☒	The statistical test(s) used AND whether they are one- or two-sided<br>*Only common tests should be described solely by name; describe more complex techniques in the Methods section.*
☐	☒	A description of all covariates tested
☐	☒	A description of any assumptions or corrections, such as tests of normality and adjustment for multiple comparisons
☐	☒	A full description of the statistical parameters including central tendency (e.g. means) or other basic estimates (e.g. regression coefficient) AND variation (e.g. standard deviation) or associated estimates of uncertainty (e.g. confidence intervals)
☐	☒	For null hypothesis testing, the test statistic (e.g. *F*, *t*, *r*) with confidence intervals, effect sizes, degrees of freedom and *P* value noted<br>*Give P values as exact values whenever suitable.*
☒	☐	For Bayesian analysis, information on the choice of priors and Markov chain Monte Carlo settings
☒	☐	For hierarchical and complex designs, identification of the appropriate level for tests and full reporting of outcomes
☒	☐	Estimates of effect sizes (e.g. Cohen's *d*, Pearson's *r*), indicating how they were calculated

Our web collection on statistics for biologists contains articles on many of the points above.

Software and code

Policy information about availability of computer code

Data collection	No software was used in data collection.
Data analysis	All analyses were performed in R v4.1.2, using custom scripts written by authors. These relied upon the following packages: phytools (v1.2-0), caper (v1.0.1). Scripts necessary for replication of all analyses and figures are attached in the submission and are uploaded to Figshare (doi:10.6084/m9.figshare.23941488). These are held privately until article publication, and at the time this report was written can be accessed through the following temporary: https://figshare.com/s/cf0fe0e5752f5205100b

For manuscripts utilizing custom algorithms or software that are central to the research but not yet described in published literature, software must be made available to editors and reviewers. We strongly encourage code deposition in a community repository (e.g. GitHub). See the Nature Portfolio guidelines for submitting code & software for further information.

Data

Policy information about availability of data

All manuscripts must include a data availability statement. This statement should provide the following information, where applicable:

- Accession codes, unique identifiers, or web links for publicly available datasets
- A description of any restrictions on data availability
- For clinical datasets or third party data, please ensure that the statement adheres to our policy

Data associated with this work will be available from Figshare (doi:10.6084/m9.figshare.23941488). The repository is being kept private until publication, but the files can be accessed using the following temporary link: https://figshare.com/s/cf0fe0e5752f5205100b

Species data were collected from the literature (Supplementary References), from the supplementary information of Pigot et al. (2020. Nature E&E, doi.10.1038/s41559-019-1070-4) and from the AVONET database (Tobias et al., 2022. Ecol.Lett., https://doi.org/10.1111/ele.13898).
Phylogenetic data were obtained from Cooney et al. (2017. Nature, doi.10.1038/nature21074).
Species richness data were obtained from Birds of the World (https://birdsoftheworld.org/bow/home).
All information and data is provided in the supplementary files.

Human research participants

Policy information about studies involving human research participants and Sex and Gender in Research.

Reporting on sex and gender	This information has not been collected.
Population characteristics	NA
Recruitment	NA
Ethics oversight	NA

Note that full information on the approval of the study protocol must also be provided in the manuscript.

Field-specific reporting

Please select the one below that is the best fit for your research. If you are not sure, read the appropriate sections before making your selection.

☐ Life sciences ☐ Behavioural & social sciences ☒ Ecological, evolutionary & environmental sciences

For a reference copy of the document with all sections, see nature.com/documents/nr-reporting-summary-flat.pdf

Ecological, evolutionary & environmental sciences study design

All studies must disclose on these points even when the disclosure is negative.

Study description	This study aimed to answer questions regarding the relationship of clade diversity with complexity of the appendicular skeleton in living birds. Estimates of species richness for extant orders were compared to mean inferred complexity of the appendicular skeleton, using a PGLS analysis applied to 34 extant orders. Further, we sought to investigate the potential influence of ecology on this correlation. Species-level data for habitat type, trophic niche, and foraging niche, were compared to species' complexity scores in PGLS analyses across 983 extant species. We also quantified the number of each of these categories which were occupied by the members of each Order, and compared these approximations of ecological specialism to both mean ordinal complexity and ordinal species richness. These were also performed using PGLS analysis. These provide a statistical description of the correlation between diversity and complexity across higher-level bird taxa, how complexity may vary between ecological niches, and how the degree of ecological specialisation exhibited by a clade relates to their mean complexity and extant species richness. A randomisation approach was used to assess the null expectation for the correlation between ecological specialisation and diversity, to provide context to the empirical results. Null distributions were generated using 1,000 random permutations of the data, assigned randomly into groups of the same number and size as the original empirical groups.
Research sample	We sought to sample the entire diversity of crown group Aves as thoroughly and evenly as was possible from published literature sources. COVID travel restrictions over much of the lifespan of this work made first hand collection visits impractical. Data from a total sample of 4023 specimens, representing 983 species in 35 orders, were collected. These represent approximately 10% of living avian diversity, and represent the vast majority of all living orders. Data for each specimen are the total lengths in millimetres of their humerus, ulna/radius, carpometacarpus, femur, tibiotarsus, and tarsometasarsus. These were chosen as they are widely available from the literature, and provide a representation of gross morphological differentiation across the limb skeleton of birds, in particular proportional differences in segment length within limb pairss, and total length differences between limb pairs.
Sampling strategy	The goal was to sample as broadly and evenly across extant avian taxonomy as possible, subject to availability of specimens described sufficiently in published literature. Literature searches to obtain these data were intended to be exhaustive (COVID travel restrictions prohibited visiting collections first hand), and have yielded a sizable data set which sample all major living groups of birds.
Data collection	Searches of published literature were performed using keyword searches in Google Scholar, and were recorded from the resulting literature sources by the corresponding author. Data were recorded as reported by the original authors of those works. 5 specimens, which are indicated in Supplementary Data 1, were collected from the University Museum of Comparative Zoology, Cambridge University, UK, by the corresponding author. This was performed using a Mitutoyo DC-8" AX (8"/200mm). Measurements were taken from each specimen three times, and the mean values reported.
Timing and spatial scale	The species sampled are globally distributed. Data collection from the literature began in the Autumn of 2020, terminating in the Summer of 2021, when literature sources began to get exhausted and the search yielded diminishing returns. As this was a literature search, frequency and periodicity of collection efforts are not relevant and do not affect experimental outcomes. The final 5 specimens consulted were housed at the University Museum of Comparative Zoology, Cambridge University, UK, visited in the Spring of 2022. These were intended to supplement underrepresented groups.

Data exclusions	Juvenile specimens were not included, as these frequently entail allometric differences in proportion which would have confounded analyses.
Reproducibility	All analyses and plotting were conducted within a single R script, which is attached in the submission and will become available in the DRYAD digital repository, pending publication.
Randomization	Species were assigned to Orders. All analyses included a phylogenetic correction, which should minimise or entirely remove the effects of phylogenetic autocorrelation upon results. In randomisation analyses, data were randoly reshuffled into groups of the same number and size as the empirical groups, to ensure that results were representative and provided accurate context.
Blinding	Blinding was not performed, as it was not necessary. The nature of this study precludes the need for blinding, as there are no participants who require blinding from each other, and no data collection or analyses entail a subjective component (e.g., scoring or classification) on the part of any of the authors. Morphological data (bone lengths, body mass, and Kipp's distance) are objective, and ecological category data were collected by other authors.

Did the study involve field work? ☐ Yes ☒ No

Reporting for specific materials, systems and methods

We require information from authors about some types of materials, experimental systems and methods used in many studies. Here, indicate whether each material, system or method listed is relevant to your study. If you are not sure if a list item applies to your research, read the appropriate section before selecting a response.

Materials & experimental systems

n/a	Involved in the study	
☒	☐	Antibodies
☒	☐	Eukaryotic cell lines
☒	☐	Palaeontology and archaeology
☒	☐	Animals and other organisms
☒	☐	Clinical data
☒	☐	Dual use research of concern

Methods

n/a	Involved in the study	
☒	☐	ChIP-seq
☒	☐	Flow cytometry
☒	☐	MRI-based neuroimaging
