# Supplementary material for: Bird clades with less complex appendicular skeletons tend to have higher species richness
Source: Nat Commun. 2023 Sep 19;14:5817. doi: 10.1038/s41467-023-41415-2 (PMC10509246; doi:10.1038/s41467-023-41415-2)
Supplement: Supplementary file 4 — Description of Additional Supplementary Files [file 41467_2023_41415_MOESM4_ESM.pdf]

Description of Additional Supplementary Files

File Name: Supplementary Data 1

Description: A .csv file containing complete information on species-level limb bone length and ecological data collected for this work.

File Name: Supplementary Data 2

Description: A document containing species richness estimates for ordinal-level taxa used in analyses. These were obtained from the Handbook of the Birds of the World (Online).

File Name: Supplementary Code 1

Description: A .zip folder containing the Supplementary_Software.R file which can be used to replicate all analyses and figures, and two tree files which are described in the Methods and utilised by the script to conduct analyses and figure production."
